# Sclerosing Mesenteritis as a Cause of Abdominal Mass and Discomfort in an Elderly Patient: A Case Report and Literature Review

**DOI:** 10.1155/2010/625321

**Published:** 2010-06-29

**Authors:** Farzana Nawaz Ali, Sidra Ishaque, Bushra Jamil, Nasir-Ud-Din, Muhammad Idris

**Affiliations:** ^1^Medical College, The Aga Khan University, (Stadium Road), Karachi 74800, Pakistan; ^2^Department of Internal Medicine, The Aga Khan University, (Stadium Road), Karachi 74800, Pakistan; ^3^Department of Pathology and Microbiology, The Aga Khan University, (Stadium Road), Karachi 74800, Pakistan; ^4^Department of Radiology, The Aga Khan University, (Stadium Road), Karachi 74800, Pakistan

## Abstract

Sclerosing mesenteritis is a rare benign process that involves inflammation, fat necrosis, and fibrosis of the mesentery. The disease poses great diagnostic challenge due to its nonspecific clinical and diagnostic findings. We report the case of a 75-year-old man who presented with vague abdominal discomfort associated with an intra-abdominal mass. With suspicion of a bowel carcinoid tumor on computed tomography scans, the patient underwent diagnostic laparoscopy. A diagnosis of sclerosing mesenteritis was made on histological examination. The patient's symptoms responded to a combination of immunosuppressive drugs, with no interval change in the size of the mass on radiological examination after fifteen months.

## 1. Introduction

Sclerosing mesenteritis is defined as a rare, benign process involving mesenteric adipose tissue [1]. Mostly affecting adults of middle age or older, it has been reported twice as often in men than in women [2]. Three types of pathological changes have been described within the mass, which include fat necrosis, chronic inflammation, and fibrosis [3, 4]. Various terms have been used to describe the condition and these include mesenteric lipodystrophy, retractile or liposclerotic mesenteritis, mesenteric Weber-Christian disease, xanthogranulomatous mesenteritis, mesenteric lipogranuloma, and systemic nodular panniculitis [4]. However, depending on the predominant histological features which include fat necrosis, inflammation, or fibrosis and retraction, the condition is referred to as mesenteric lipodystrophy, panniculitis or retractile mesenteritis, respectively [4]. Due to varied and non-specific presentations and findings, the disease poses great diagnostic challenges leading to misdiagnosis in majority of the cases. Although definitive diagnosis requires biopsy and histopathology, various radiological modalities such as multidetector CT and MRI have been used to make the diagnosis with appropriate clinical analysis. 

We report a case of a 75-year-old gentleman, who presented with vague abdominal discomfort and an intra-abdominal mass. Secondary to a strong suspicion of a bowel carcinoid tumor on abdominal CT scan, he underwent a diagnostic laparoscopy and minilaparotomy. A diagnosis of sclerosing mesenteritis was made on histopathology and the patient was started on an immunosuppressive regimen.

## 2. Case Presentation

**Figure 1:**
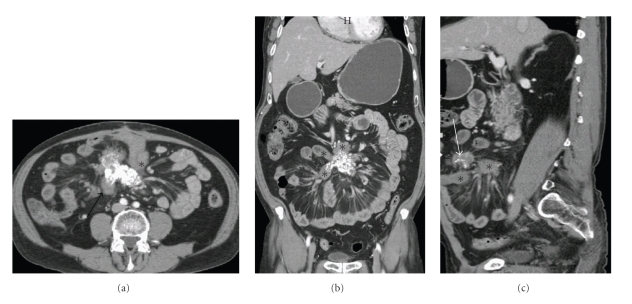
*Sclerosing mesenteritis*. (a) Contrast enhanced CT Axial section through the mid abdomen shows a heterogeneous soft tissue density (black arrow) with calcifications in the root of mesentery and mesenteric fat stranding with fibrotic kinking and traction over small bowel loops as shown by an asterisk. (b) Contrast enhanced CT coronal section redemonstrating fibrosing reaction in the mesentery with pulling of adjacent bowel loops. (c) Contrast enhanced CT sagittal section clearly showing speculation in the mesenteric mass (straight white arrow) with mesenteric fat infiltration.

A 75 years old male patient, presented to the outpatient department with a 3-month history of vague abdominal discomfort (stretching sensation in the upper abdomen) and an intra-abdominal mass “about the size of his fist” which he believed had gradually increased in size over the previous 2 months. On examination the mass was found to be located above the umbilicus; it was mobile, smooth, approximately 6 × 7 × 5 cm and slightly tender on deep palpation. The baseline laboratory workup consisted of routine blood tests including hepatic and renal function tests which were all unremarkable. An abdominal CT scan with IV contrast revealed a heterogeneous, soft tissue, mesenteric mass with calcifications in the root of the mesentery, encasing the superior mesenteric vessel. Mesenteric fibrosis resulting in tethering of small bowel loops with extensive desmoplastic reaction and small mesenteric lymph nodes were also noted (Figures 1(a), 1(b), and 1(c)). 

With high suspicion of bowel carcinoid tumor by the radiologist (although a differential diagnosis of fibrosing mesenteritis was also suggested), a diagnostic laparoscopy was performed. Intraoperatively, the mass was found to be located in the paraaortic region, at the root of mesentery. Because the friable lesion bled easily on touch, the procedure was converted to a minilaparotomy. 

**Figure 2:**
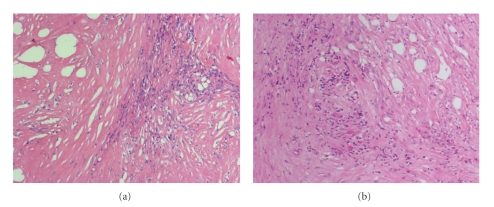
(a) (H&E, magnification 10×). Dense collagen with fascicles of bland fibroblasts. (b) (H&E, magnification 10×). Collagen bundles with associated fibroblasts and chronic inflammatory cells.

A wedge of the mass was taken for histopathology examination which revealed extensive fibrosis along with a few areas of necrosis. Occasional collections of histiocytes were noted, but no evidence of granuloma or malignancy was identified (Figures 2(a) and 2(b)).

 The diagnosis of sclerosing mesenteritis was made and the patient was started on a regimen of immunosuppressive drugs which included prednisolone and azathioprine, along with tamoxifen. Over the past 30 months, the patient has been on tamoxifen and azathioprine. Tamoxifen has been shown to be useful in the treatment of desmoid tumors and idiopathic retroperitoneal fibrosis [5]. Another reason for combining tamoxifen with the immunosuppressive regimen was that this drug is relatively safe and simple to dose.

Prednisolone has been tapered off and restarted twice over this time, depending on the symptoms which include feeling of stretch in the abdomen and lack of appetite. Follow-up CT scans of the abdomen at 2, 6, 8, and 15 months after the biopsy revealed no interval change in the appearance of the mass. The patient had been on the specified regimen until he developed herpes zoster along the left mandibular division of the trigeminal nerve. This required stopping azathioprine. However, over a period of 6 weeks, this led to worsening of symptoms and both steroids and azathioprine had to be restarted. Currently, he is stable clinically and his steroids are being tapered off.

## 3. Discussion

Sclerosing mesenteritis (SM) is a rare, benign, and chronic inflammatory disorder of the small and large bowel mesentery. Rarely, it may affect the peripancreatic region, omentum, retroperitoneum, and pelvis [2, 4]. The vast majority of the cases are idiopathic, however, some cases have been found to be associated with autoimmunity, ischemia, infection, vasculitis, Gardner's syndrome, carcinoid, trauma, previous abdominal surgery, and various cancers [6–10]. 

The clinical manifestations are largely non-specific. The patient may be absolutely asymptomatic with diagnosis made incidentally. Other findings may include, vague abdominal discomfort and a palpable mass (as in the case discussed), distention, progressive fatigue, weight loss, nausea and vomiting, tender mass in the abdomen, fever, malabsorption syndrome, chylous ascites, and pneumoperitonium [2, 4, 6, 10–12]. Laboratory workup such as complete blood count, peripheral blood film, erythrocyte sedimentation rate, serum C-reactive protein, amylase, lipase, liver and renal function tests, and autoimmune workup is usually negative in cases of pure sclerosing mesenteritis [4, 13, 14] as was observed in our patient. 

CT findings associated with sclerosing mesenteritis are variable. It may be seen as a subtle increased attenuation to a solid soft-tissue mass in the small bowel mesentery. Other findings include, enveloping of and preservation of fat around the mesenteric vessels, a phenomenon that is referred to as the “fat ring sign” [15]. Several conditions can mimic the CT appearance of sclerosing mesenteritis, including lymphoma, carcinoid tumor, carcinomatosis, primary mesenteric mesothelioma, mesenteric fibromatosis, and mesenteric edema. In the case described, the patient's initial CT scan was suggestive of a sclerosing mesenteritis. The inability to distinguish it from a carcinoid tumor of the bowel warranted obtaining a tissue sample for histopathological study [16]. Although CT and MRI scans may suggest the diagnosis of sclerosing meseneteritis, the definitive diagnosis requires biopsy and histological evaluation as it is imperative to exclude an underlying infection or malignancy.

No generalized consensus has been reached so far regarding the management of sclerosing mesenteritis. It may be difficult to distinguish it from other bowel diseases and tumors preoperatively on radiological scans. Intraoperatively, repetitive frozen section examinations may reveal nonspecific inflammatory changes alone. Therefore, due to the possibility of misdiagnosis preoperatively and intraoperatively, it is sometimes required to remove the mass surgically in order to avoid repetitive operations [14]. 

Surgery may also be attempted if medical therapy fails or in the presence of life threatening complications such as bowel obstruction or perforation [17]. In cases of colonic involvement by sclerosing mesenteritis, a colostomy may be necessary because complete surgical resection is often not technically possible. However, the surgical approach is often limited by vascular involvement [18].

Watchful waiting alone may suffice in cases where fat necrosis is the main feature [11]. However, in cases where features of chronic inflammation predominate, several therapeutic agents have shown promising results. These include cyclophosphamide [19] along with combined corticosteroids and tamoxifen [2], and combined corticosteroids and azathioprin [20]. A 30 months follow up of our patient reveals good therapeutic outcome on the stated immunosuppressive regimen in terms of no increase in the size of the mass. However, once the mass acquires an aggressive course with progressive fibrosis and complications such as shortening of mesentery, intestinal obstruction, perforation and vascular compromise, complete surgical resection of the diseased mesentery becomes mandatory [11, 21]. 

Kipfer et al. reports [22] a coexistent malignant lymphoma in 8 of the 53 patients ultimately. The co existence of lymphomas has also been reported by others [23], justifying the clinical importance of the early diagnosis of this condition. The overall prognosis of sclerosing mesenteritis is usually very good with a benign and self limiting course in most of the cases [1–3]. The pain disappears in 75% patients and mass regresses in 66%, usually within 2 years [24].

## 4. Conclusion

Our case illustrates that the diagnosis of sclerosing mesenteritis can be difficult preoperatively. Tissue diagnosis is absolutely essential to avoid misdiagnosing a malignancy as sclerosing mesenteritis on radiological appearance. Judicious use of immunosuppressive therapy can limit progression of the condition, delaying the need for surgery.

##  Consent

Written informed consent was obtained from the patient for publication of this case report and accompanying images. A copy of the written consent is available for review by the Editor-in-Chief of this journal.

##  Competing Interests

The authors declare that they have no competing interests.

##  Authors' Contributions

F. N. Ali conceived the idea of the manuscript, and was a major contributor in data collection and writing the manuscript. S. Ishaque participated in the data collection and sequencing and drafting of the manuscript. B. Jamil was involved in direct clinical care of the patient, therapeutic planning, interpretation of the patient data and providing overall supervision in the project. N.-U. Din was involved in the interpretation of histopathology slides. M. Idris was involved in the interpretation of the CT scan. All authors read and approved the final manuscript.

## Abbreviations

SM: Sclerosing Mesenteritis
CT: Computed tomography
MRI: Magnetic resonance imaging
IV: Intravenous
H&E: Hematoxylin and Eosin stain.

## References

[1] White B, Kong A, Chang A-L (2005). Sclerosing mesenteritis. *Australasian Radiology*.

[2] Akram S, Pardi DS, Schaffner JA, Smyrk TC (2007). Sclerosing mesenteritis: clinical features, treatment, and outcome in ninety-two patients. *Clinical Gastroenterology and Hepatology*.

[3] González Rodrígez CI, Cires M, Rubio T, Jiménez FJ, García de Eulate I, Artondo MT (2008). A case of mesenteric panniculitis. *Anales del Sistema Sanitario de Navarra*.

[4] Vettoretto N, Diana DR, Poiatti R, Matteucci A, Chioda C, Giovanetti M (2007). Occasional finding of mesenteric lipodystrophy during laparoscopy: a difficult diagnosis. *World Journal of Gastroenterology*.

[5] Ergun I, Keven K, Canbakan B, Ekmekci Y, Erbay B (2005). Tamoxifen in the treatment of idiopathic retroperitoneal fibrosis. *Int Urol Nephrol*.

[6] Durst AL, Freund H, Rosenmann E, Birnbaum D (1977). Mesenteric panniculitis: review of the literature and presentation of cases. *Surgery*.

[7] Cuff R, Landercasper J, Schlack S (2001). Sclerosing mesenteritis. *Surgery*.

[8] Sharma V, Martin P, Marjoniemi V-M (2006). Idiopathic orbital inflammation with sclerosing mesenteritis: a new association?. *Clinical and Experimental Ophthalmology*.

[9] Han SY, Koehler RE, Keller FS (1986). Retractile mesenteritis involving the colon: pathologic and radiologic correlation (case report). *American Journal of Roentgenology*.

[10] Emory TS, Monihan JM, Carr NJ, Sobin LH (1997). Sclerosing mesenteritis, mesenteric panniculitis and mesenteric lipodystrophy: a single entity?. *American Journal of Surgical Pathology*.

[11] Chawla S, Yalamarthi S, Shaikh IA, Tagore V, Skaife P (2009). An unusual presentation of sclerosing mesenteritis as pneumoperitoneum: case report with a review of the literature. *World Journal of Gastroenterology*.

[12] Arora M, Dubin E (2008). A clinical case study: Sclerosing mesenteritis presenting as chylous ascites. *MedGenMed Medscape General Medicine*.

[13] Zafar AM, Rauf MA, Chawla T, Khanda G (2008). Mesenteric panniculitis with pedal edema in a 33-year-old Pakistani man: a case report and literature review. *Journal of Medical Case Reports*.

[14] Gu GL, Wang SL, Wei XM, Ren L, Li DC, Zou FX (2008). Sclerosing mesenteritis as a rare cause of abdominal pain and intraabdominal mass: a case report and review of the literature. *Cases Journal*.

[15] Horton KM, Lawler LP, Fishman EK (2003). CT findings in sclerosing mesenteritis (panniculitis): spectrum of disease. *Radiographics*.

[16] Hamrick-Turner JE, Chiechi MV, Abbitt PL, Ros PR (1992). Neoplastic and inflammatory processes of the peritoneum, omentum, and mesentery: diagnosis with CT. *Radiographics*.

[17] Issa I, Baydoun H (2009). Mesenteric panniculitis: various presentations and treatment regimens. * World Journal of Gastroenterology*.

[18] Durst AL, Yarom R, Luttwak EM (1971). Malignant fibromatous peritoneal mesothelioma associated with liposclerotic mesenteritis. *American Journal of Gastroenterology*.

[19] Bush RW, Hammar SP, Rudolph RH (1986). Sclerosing mesenteritis. Response to cyclophosphamide. *Archives of Internal Medicine*.

[20] Bala A, Coderre SP, Johnson DRE, Nayak V (2001). Treatment of sclerosing mesenteritis with corticosteroids and azathioprine. *Canadian Journal of Gastroenterology*.

[21] Nobili C, Degrate L, Caprotti R (2009). Extensive sclerosing mesenteritis of the rectosigmoid colon associated with erosive colitis. *Gastroenterology Research and Practice*.

[22] Kipfer RE, Moertel CG, Dahlin DC (1974). Mesenteric lipodystrophy. *Annals of Internal Medicine*.

[23] Durst AL, Freund H, Rosenmann E, Birnbaum D (1977). Mesenteric panniculitis: review of the literature and presentation of cases. *Surgery*.

[24] Reske M, Namiki H (1975). Sclerosing mesenteritis. Report of two cases. *American Journal of Clinical Pathology*.

